# Use of Benzodiazepines in Alzheimer’s Disease: A Systematic Review of Literature

**DOI:** 10.1093/ijnp/pyv055

**Published:** 2015-05-19

**Authors:** Michaela Defrancesco, Josef Marksteiner, W. Wolfgang Fleischhacker, Imrich Blasko

**Affiliations:** Memory Clinic (Dr Defrancesco), and Division of General and Social Psychiatry (Drs Fleischhacker and Blasko), Department of Psychiatry and Psychotherapy, Innsbruck Medical University, Innsbruck, Austria; Department of Psychiatry and Psychotherapy A, Landeskrankenhaus Hall, Hall, Austria (Dr Marksteiner); Division of Biological Psychiatry, Department of Psychiatry and Psychotherapy, Innsbruck Medical University, Innsbruck, Austria (Dr Fleischhacker).

**Keywords:** Benzodiazepines, Alzheimer’s disease, behavioral and psychological symptoms of dementia, sleep disorder, agitation

## Abstract

**Background:**

Benzodiazepines are frequently prescribed in patients with Alzheimer’s disease. Unfortunately, studies evaluating their benefits and risks in these patients are limited.

**Methods:**

Clinical trials focusing on the effect of benzodiazepines on cognitive functions, disease progression, behavioral symptoms, sleep disturbances, and the general frequency of benzodiazepine use were included in this review. Published articles from January 1983 to January 2015 were identified using specific search terms in MEDLINE and PubMed Library according to the recommendations of The Strengthening the Reporting of Observational Studies in Epidemiology initiative.

**Results:**

Of the 657 articles found, 18 articles met predefined selection criteria and were included in this review (8 on frequency, 5 on cognitive functions, 5 on behavioral and sleep disturbances). The frequency of benzodiazepine use ranged from 8.5% to 20%. Five studies reported accelerated cognitive deterioration in association with benzodiazepine use. Two studies reported clinical efficacy for lorazepam and alprazolam to reduce agitation in Alzheimer’s disease patients. No evidence was found for an improvement of sleep quality using benzodiazepines.

**Conclusion:**

This systematic review shows a relatively high prevalence of benzodiazepine use but limited evidence for clinical efficacy in Alzheimer’s disease patients. However, there is a paucity of methodologically high quality controlled clinical trials. Our results underscore a need for randomized controlled trials in this area.

## Introduction

Recently, a number of studies have examined the possible links between Benzodiazepines (BZD) use and Alzheimer’s disease (AD). Findings from retrospective studies suggest that cumulative exposure to BZD longer than 3 month may increase dementia risk (Lagnaoui et al., 2002; Billioti de Gage et al., 2014). Particularly, the use of BZD with a long half-life may be most harmful by limiting the cognitive reserve capacity (Billioti de Gage et al., 2012). AD is the most common cause of dementia among the elderly. It is estimated that the number of patients with AD will triple by 2050 to more than 115 million cases worldwide (Alzheimer’s, 2013). Although cognitive deficits are the clinical hallmark of AD, various noncognitive symptoms termed behavioral and psychological symptoms of dementia (BPSD) are common and can dominate disease presentation (Lyketsos et al., 2000, 2002). BPSD have been observed in up to 60% to 98% of patients with dementia and include agitation, aggression, anxiety, delusions, sleep disturbances, and hallucinations among other symptoms (Mega et al., 1996; Margallo-Lana et al., 2001). To date, there is still disagreement on the use of BZD as an alternative to antipsychotics for treating BPSD. However, BZD are widely prescribed to control disruptive behavior and sleep disturbances in AD patients (Hoiseth et al., 2013). Earlier studies in healthy elderly and AD patients have suggested some effectiveness, particularly of those with short half-lifes (eg, oxazepam), in treating agitation and aggressive symptoms (Gerz, 1964; Sanders, 1965).

Although good practice guidelines recommend a limited duration of BZD prescription to a few weeks, their use is often chronic and most patients take them for years (American Geriatrics Society Beers Criteria Update Expert, 2012). Long-term treatment with BZD has been associated with an increased risk of falls, dependence, and withdrawal syndromes (Voyer et al., 2010), yet in dementia patients the risk of falls was reported to be slightly more frequent due to antidepressants and antipsychotics (Sterke et al., 2012). Moreover, prior studies in AD patients suggest that BZD worsen cognitive impairment, and lead to a higher rate of side effects such as amnesia, confusion, sedation (Petrovic et al., 2003; Madhusoodanan and Bogunovic, 2004), as well as other adverse drug reactions (ADRs) (Ingum et al., 1994; Allain et al., 2005). The prevalence of such ADRs in patients with AD is estimated to be between 5% and 10% (Onder et al., 2002; Hogan et al., 2003; Laroche et al., 2013).

The current literature suggests that the high rate of BZD-induced side effects in AD is based on disease related changes of pharmacokinetics, pharmacodynamics, and the neurotransmitter system. BZD actions are mediated via gamma-aminobutyric acid A (GABA _A_) receptors. In contrast to the marked deficits seen in cholinergic and glutamatergic systems in AD brains, the inhibitory GABAergic pathway appears to be more resistant to neurodegeneration and relatively spared (Rissman et al., 2007). This preservation of GABAergic neurons could even support the use of BZD in AD patients. In contrast, radioligand studies have demonstrated a reduction of GABA _A_-receptor binding sites as well as their function in the frontal and temporal cortices of AD brains (Shimohama et al., 1988;Sasaki et al., 1986; Mizukami et al., 1997). Although prior studies suggest GABAergic remodeling in the brain and an age-dependent reduction of GABA currents (Limon et al., 2012), little is known about the functionality of the GABA _A_ receptor in AD. Evidence from clinical observations suggests that BZD might enhance cognitive decline (Larson et al., 1987; Lopez et al., 1999) and possibly exacerbate steps specific for neurodegeneration in AD, for example, by increasing intraneuronal Aβ42 accumulation as shown in a transgenic mice model (Tampellini et al., 2010). However, the findings of experimental studies do not allow for definitive conclusions at this time.

In summary, numerous studies have studied the effect of BZD in the healthy elderly and dementia patients, but results are inconsistent and allow no valid recommendation for the appropriate usage of BZD in patients with AD. Furthermore, studies are often limited by an insufficient description of the study population and fail to provide a critical look deeper into specific substances, dosages, and indications.

We have therefore reviewed the existing literature regarding the use of BZD in patients with AD. We concentrated on high-quality studies, which focus on the effect of BZD on cognitive functions, disease progression, behavioral symptoms, sleep disturbances, and the general frequency of BZD use. All included studies examined BZD use in patients who underwent an established diagnostic setup for AD and provided evidence-based assessments of risks and benefits of BZD in these patients.

## Methods

For the purpose of this systematic review, we followed the revised PRISMA guidelines, which have been updated to address several conceptual and practical advances in the science of systematic reviews (Moher et al., 2009). Two independent authors (I.B. and M.D.) undertook the literature search, assessed eligibility, and summarized results. Any discrepancies during these processes were resolved through consensus oriented discussions.

### Search

Online published articles from January 1983 to January 2015 were searched using the PubMed/MEDLINE database. To build up the literature search in PubMed, we used the following MeSH-terms (Medical Subject Headings, controlled vocabulary thesaurus used for indexing articles): “Alzheimer Disease”[Mesh] AND “Benzodiazepines,”[Mesh] “Alzheimer Disease”[Mesh] AND “Hypnotics and Sedatives,”[Mesh] “Alzheimer Disease”[Mesh] AND “Anti-anxiety agents,”[Mesh] and “Alzheimer Disease”[Mesh] AND “Tranquilizing Agents.” Limits were set for: “language English,” “humans,” “full text available,” and “clinical trial.” Furthermore, unpublished studies were searched in the clinical trial registries (http://clinicaltrials.gov/). Additionally, reference lists of relevant articles and related citations found by electronic search were hand-searched for the identification of additional articles using the same search terms as mentioned above.

### Selection Criteria

Studies were selected based on the following inclusion criteria: (1) original research papers with prospective or retrospective design (double- blind, placebo-controlled or randomized controlled trials [RCTs], observational studies, population-based studies, cohort studies), (2) AD patients had to be diagnosed according to one of the following diagnostic criteria: the Diagnostic and Statistical Manual of Mental Disorders (DSM) in its third or fourth edition, the International Classification of Diseases in its tenth edition, the National Institute of Neurological and Communicative Disorders and Stroke-AD and Related Disorders Association (NINCDS-ADRDA) criteria (McKhann et al., 1984) or its revised version (Dubois et al., 2007), English language, (4) carried out in humans, (5) published in an peer-reviewed journal, and (6) full text available.

Studies were excluded in case of: (1) missing information regarding applied diagnostic procedure of dementia, (2) dementia was diagnosed based on measures of primary sociobiological functions (Katz-criteria) (Katz and Akpom, 1976), or (3) detection of symptoms of psychological distress, not fulfilling the above mentioned diagnostic criteria of dementia (eg, Helmes et al., 1987).

### Outcome Parameters

The 2 primary outcome parameters of this systematic review were: (1) the frequency of BZD use, and (2) the effects of BZD on cognition functions as well as behavioral and psychological symptoms of dementia, including sleep disturbances in patients with AD or mixed dementia. As secondary outcomes, we extracted information on the following: type of BZD (dose, formulation method), dementia severity, sample size, study duration, and type of study population.

## Results

**Figure 1.**
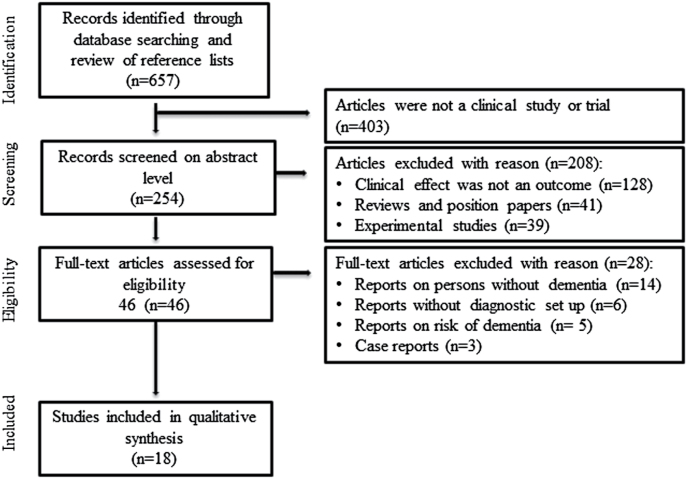
Flow chart with detailed stepwise selection procedure and reasons for exclusions.

A total of 657 articles were identified through the PubMed/MEDLINE database search and assessed for eligibility. Figure 1 shows the stepwise selection procedure and reasons for exclusions. Of 657 identified reports, 18 articles met the predefined inclusion criteria and were included in the review. The articles were divided into 3 categories: (1) frequency of BZD-use in persons with AD, (2) effect of BZD on cognitive functions, and (3) BZD for BPSD and sleep disturbances in AD.

### Frequency of BZD Use in Persons with AD

We identified 8 eligible studies assessing the frequency of BZD use in patients with AD or mixed dementia. As presented in Table 1, sample size varied from 66 (Steve et al., 2008) to 4.214 patients (Lagnaoui et al., 2003; Koyama et al., 2013; Montastruc et al., 2013). Table 2 provides information on study designs and main findings associated with BZD use. Five studies were designed as prospective and 3 as cross-sectional. As can be seen from Table 5, of these 8 studies, 3 included exclusively AD patients with mild to moderate stages of the disease (MMSE 21–26), reporting a frequency of BZD use ranging from 8.5% (Montastruc et al., 2013) to 20% (Balfour and O’Rourke, 2003; Lagnaoui et al., 2003). In the largest cohort (n = 4.214), examined by Lagnaoui et al. (2003), a MMSE score <24 was associated with less frequent BZD use. A high level of BZD use correlated with high levels of overall drug consumption. Similarly, Steve et al. (2008) reported a positive correlation of low MMSE scores and BZD intake in patients from memory clinics (Steve et al., 2008).

**Table 1.** General Study Characteristics

	Population	AD Cases Only	Diagnostic Criteria	Dementia Stage	Age (mean y)	Study Duration (y)	Sample Size
Frequency of BZD Use							
Koyama et al., 2013	Community-dwelling older women	n.d.	NINCDS-ADRDA	MMSE 26.5	87.6	10	1.484
Montastruc et al., 2013	Participants living at home	Yes	NINCDS-ADRDA DSM- IV	CDR 1.1±0.6	77.9	4	684
Sterke et al., 2012	Nursing home residents	n.d.	DSM-IV-TR	GDS 5–6	82	2	284
Wetzels et al., 2011	Nursing home residents	No	NINCDS-ADRDA	GDS 6–7	81.7	2	117
Nobili et al., 2009	Residents of special care units	No	Clinical diagnosis	MMSE 7.8±7.0	81.2	1.5	349
Steve et al., 2008	Memory clinic	No	Clinical diagnosis	MMSE 21.6±7.0	74.1	n.a.	66
Balfour et al., 2003	Community dwelling and institutionalized patient	Yes	NINCDS-ADRDA	3MS 0–77	84.3	n.a.	460
Lagnaoui et al., 2003	Community- dwelling and institutionalized patients	Yes	NINCDS-ADRDA DSM- III-R	MMSE>24 in >90% cases	73.2	0.25	4.214
BZD effect on cognitive functions							
Rosenberg et al., 2012	Community- dwelling and institutionalized patient	Yes	NINCDS-ADRDA	CDR 5.5*	86.6	3.7 mean	230
Ellul et al., 2007	Community- dwelling, nursing homes/primary care	Yes	NINCDS-ADRDA	GDS 4–5	82.3	1	257
Lopez et al., 1999	Dementia research clinic	Yes	NINCDS-ADRDA	MMSE 16–19	71.3	4.2 mean	179
Sunderland et al., 1989	Not mentioned	Yes	NINCDS-ADRDA DSM- III	GDS mild, moderate	60.1	1 and 2h post drug	20
Larson et al., 1987	Mixed population	No	DSM-III	MMSE 19.1±7.9	77.1	1	308
BZD use for behavioral and sleep disturbances							
Liao et al., 2012	Dementia specialty care unit	Yes	Revised NINCDS- ADRDA	CDR 2	59–85	7 days	7
Meehan et al., 2002	Inpatients	n.d.	NINCDS-ADRDA or DSM IV	MMSE 11.8±7.1	77.6	24 h	272
McCarten et al., 1995	Outpatient men from memory clinic	Yes	NINCDS-ADRDA DSM IV	MMSE 11.6±6.4	73	8 days	7
Ancill et al., 1991	Psychogeriatric Inpatient Unit	No	DSM-III-R	CGI	78.9	28 days	40
Coccaro et al., 1990	Chronic care ward	No	DSM-III, NINCDS- ADRDA	CDR 3	75.3	8 weeks	52

Abbreviations: CDR, Clinical Dementia Rating scale; CGI, Clinical Global Impression; GDS, Global Deterioration Scale; MMSE, Mini-Mental State Examination; 3MS Modified Mini-Mental State Examination; NINCDS-ADRDA, National Institute of Neurological and Communicative Disorders and Stroke-AD and Related Disorders Association; n.a., not applicable; n.d., not determined.

*Represents CDR-sum score.

Table 1 gives information on general characteristics of included studies in this review. As can be seen from the table, there was a high variability of sample size and study duration across the reviewed studies.

**Table 2.** Methodological Characteristics of Included Studies Assessing the Frequency of BZD Use

	Study Design	Methodology Aim, data acquisition, definition of BZD exposure	Main Findings Associated with BZD Use	Adverse effects of BDZ
Koyama et al., 2013	Prospective cohort	PIM use according to Beers criteria over 10 years in female participants recruited from study on osteoporosis bringing all their medication to each visit. At 10-years a panel of clinical experts adjudicated cognitive status.	At 10 years, BZDs were the second common PIM (8.6%); overall PIM use increased for women with dementia (24.9–33.1%) but remained fairly constant for MCI –patients (23.9–23.0%).	n.d.
Montastruc et al., 2013	Prospective cohort	Use of PIM according to Laroche and Beers criteria over 4 years in patients recruited from a university hospital-based network of interdisciplinary teams.	8.5 % of AD patients used long half-life BZD. Female gender and polypharmacy was associated with higher PIM use.	n.d.
Sterke et al., 2012	Observational cohort	To study the magnitude of associations between fall risk and psychotropic drugs (incl. BZDs) use. Database on daily drug use and daily falls in nursing home was established and dose-response relationship evaluated.	20.9% of anxiolytics and 13.6% hypnotics expressed as person-days were used.	Increased fall risk for anxiolytics (HR 1.6) and hypnotics (1.5).
Wetzels et al., 2011	Prospective cohort	Patterns of psychotropic drug use across dementia subtypes in nursing homes. Data on psychotropic drug use was retrieved from the patients’ medical and pharmacist files.	Anxiolytic and hypnotics were used continuously in 3.4% and 9.4% resp. of residents with different prescription patterns among residents with AD and vascular dementia.	n.d.
Nobili et al., 2009	Prospective observational	Prevalence of antipsychotic use and their association with BPSD and other clinical and epidemiological predictors. Data were collected by a trained physician of the special care unit staff.	40% patients on antipsychotics medication received additional BZD, which in 85% of cases was a short-acting agent.	n.d.
Steve et al., 2008	Cross-sectional	Assesses the extent of anticholinergic and BZD medication use in AD patients versus patients without AD. Caregivers were asked to bring patients’ medications to appointment.	14% of consecutively assessed patients of memory clinic used BZDs.	n.d.
Balfour et al., 2003	Cross-sectional	To study if analgesia could be underutilised among patients with AD. Prescription of analgesics and psychotropic medication for AD patients with and without musculoskeletal conditions. Information on chronic health conditions and medication was obtained from primary caregivers.	AD patients with arthritis or rheumatism were more likely to be prescribed BZD compared to AD patients without musculoskeletal conditions.	n.d.
Lagnaoui et al., 2003	Cross-sectional	To assess patients and drug- characteristics associated with BZD use. BZD use within 3 months from medical records and by informant.	20% of AD patients were BZD users. Low MMSE was associated with a decreased BZD use.	n.d.

Abbreviations: AD, Alzheimer’s disease; BZD, benzodiazepine; MMSE-Mini-Mental State Examination, PIM, potentially inappropriate medication, n.d., not determined.

Table 2 presents used study designs, methodology, and main findings of the 8 included studies, which assessed the frequency of BZD use in patients with AD.

Five studies assessed BZD as Potentially Inappropriate Medication (PIM) in a mixed population of institutionalized patients in nursing homes or care facilities with mild cognitive impairment, severe AD, or mixed dementia (Nobili et al., 2009; Wetzels et al., 2011; Sterke et al., 2012; Koyama et al., 2013; Montastruc et al., 2013). Koyama et al. (2013) analyzed data from a 10-year follow-up study conducted in community-dwelling women with mild to severe cognitive impairment (Table 2). In this study, BZD were the second most prescribed PIMs with a frequency of 8.6%. Overall, percentage of usage of PIMs including BZD showed an increase from 23.9% to 33.1% for women with dementia but remained fairly constant women with no (22.2–19.8% PIMs) or mild cognitive impairment (23.9–23.0% PIMs).

A cohort study by Sterke et al. (2012) found that BZD were the second or third most prescribed psychotropic drugs in nursing home residents. Oxazepam and temazepam were used with highest frequency. In this population, the intake of BZD as well as the combination of BZD with any other psychotropic medication was associated with a significant increase of fall risk in a dose-dependent relationship (Sterke et al., 2012). Wetzels et al. (2011) reported that 9.4% of nursing home residents were treated with BZD continuously for 2 years in another prospective cohort study. BZD along with antidementive medication were more frequently prescribed for residents with AD, whereas residents with vascular dementia more often received antipsychotics, antidepressants, and anticonvulsants.

Nobili et al. (2009) investigated the use of psychotropic drugs among patients with BPSD treated in an Alzheimer special care unit. Approximately 24% of all patients received BZD. Of thesem 40% were additionally treated with antipsychotics (Table 3). The most frequent coadministered BZD were lorazepam (35%), triazolam (16%), oxazepam (10%), and diazepam (10%). In a French cohort of 684 outpatients with mild to moderate AD (MMSE 10–26), only 8.5% received long-acting BZD (Montastruc et al., 2013).

**Table 3.** Methodological Characteristics of Included Studies Assessing BZD Effect on Cognitive Functions

	Study Design	Methodology Aim, data acquisition, definition of BZD exposure	Main Findings Associated with BZD Use	Adverse effects of BDZ
Rosenberg et al., 2012	Longitudinal population- based	Association of antipsychotics/BZDs with cognitive, functional, and neuropsychiatric outcome. Medication exposure measured by calculation of a PI. Data obtained from inspection of medication vials and medical records brought to investigation.	Higher PI for antipsychotics/ BZDs positively correlated with increased dementia severity (CDR), higher PI of BZD with a more rapid decline in cognition (MMSE).	n.d.
Ellul et al., 2007	Longitudinal cohort	Effect of medications (eg, antipsychotics, antidepressants, hypnotics, anxiolytics, and antidementives) on the rate of disease deterioration defined as increase of one point in GDS.	BZD alone (OR 2.7) and combined with antipsychotics (OR 3.9) showed a faster rate of deterioration in GDS	n.d.
Lopez et al., 1999	Longitudinal follow-up	Assessment of the predictive value of psychiatric medication and behavioural disturbances on progression of AD. Likelihood of arriving at 4 predefined clinical end points: worsening of cognition, ADL, admission to nursing home and death.	BZD use was associated with shorter time to death (RR 2.0).	n.d.
Sunderland et al., 1989	Double-blind, placebo- controlled	Determine the attentional, learning, and memory effects of a centrally active low-dose BZD (lorazepam 1mg) in 10 patients with AD and 10 age matched controls. Neuropsychological tests were performed at baseline and repeated at 1h and 2h postdrug administration.	Lorazepam increased impairment in the continuous performance task and attention but decreased restlessness of AD patients.	Higher rate of drowsiness and dry mouth,, lowering of blood pressure
Larson et al., 1987	Prospective observational	Influence of ADRs caused by psychotropic medication, ie, BZDs, on cognitive impairment. Occurrence of predefined ADRs was classified as: definite, probable, possible or unlikely	Longer-acting BZDs were the commonest drug associated with cognitive impairment.	BZD increased risk for ADRs to 5.9 times, use of sedatives together with hypertensives increase risk of falls

Abbreviations: AD, Alzheimer’s disease; ADL, activities of daily living; ADR, adverse drug reaction; BZD, benzodiazepine; CDR, Clinical Dementia Rating scale; GDS, Global Deterioration Scale; MMSE-Mini-Mental State Examination; PI, persistency index; n.d., not determined; OR, Odds Ratio.

Table 3 summarizes the study designs, methodology, and main findings of the 5 included studies, which assessed the effect of BZDs on cognitive functions in patients with AD.

Balfour and O’Rourke (2003) addressed the important aspect of administering BZD for the treatment of pain, misinterpreted as dementia-related behavior. They showed that BZD were more commonly prescribed for AD patients with arthritis or rheumatism compared with those without these diseases (22% vs 16%).

### Effect of BZD on Cognitive Functions

We identified 5 studies assessing the effect of BZD on cognition functions in AD patients (Sunderland et al., 1989; Lopez et al., 1999; Ellul et al., 2007; Rosenberg et al., 2012). As shown in Table 5, 4 of 5 studies included exclusively patients suffering from AD. Rosenberg et al. (2012) examined the association of psychotropic medication use with cognitive, functional, and neuropsychiatric symptom in 224 patients with probable AD during a 12-month period. The effect of multiple drugs including BZD was examined by the Global Deterioration Scale. Longer duration of exposure to psychotropic medication, including BZD, was associated with a more rapid decline in cognition (MMSE) and increase in dementia severity (CDR, NPI). Furthermore, BZD users compared with nonusers showed a 2.8 times higher deterioration rate on the Global Deterioration Scale score.

**Table 4.** Methodological Characteristics of Included Studies Assessing the Effect of BZDs for Behavioral and Sleep Disturbances

	Study Design	Methodology Aim, data acquisition, definition of BZD exposure	Main Findings Associated with BZD Use	Adverse effects of BDZ
Liao et al., 2012	Retrospective analysis of prospectively collected case series	Effect of brotizolam, single dose 0.25mg on nocturnal and daily activities assessed by a 24-h circuit tag monitoring system.	increase incidence of nighttime wandering and day-time restlessness (reversed rest-activity patterns)	n.d.
Meehan et al., 2002	Multicenter, randomized, double- blind, placebo-controlled	Effect of either olanzapine 2.5mg or olanzapine 5.0mg or lorazepam 1.0mg or placebo on agitation at 30, 60, 90, 120min, and 24 hours after the intra muscular (i.m.) administration. Changes were measured on panel of neuropsychiatric scales.	I.m. injection of 2.5mg or 5.0mg olanzapine or 1.0mg lorazepam significantly improved agitation. Olanzapine 5mg showed fastest onset.	No significant adverse events in the treatment groups.
McCarten et al., 1995	Open study, placebo - drug - placebo	Effect of triazolam on total sleep time and memory function was tested in an intermediate care ward. Patient with AD received triazolam 0.125mg and were followed by portable wrist activity monitor for 8 days.	No effect on sleep time and recent memory deficits	No side effects concerning sleep and memory related to BZD, risk of falls n.d.
Ancill et al., 1991	Randomized, double-blind comparison	Comparison of alprazolam 0.25mg to lorazepam 0.5mg 3 times/d for treating agitation. Between days 1 and 28, stepwise dosage adjustments were made. Symptoms were assessed on day 0, 7, 14, 28; Physicians Global Impression and ADRs were recorded.	Nonstatistical signitficant improvement in agitation of 29% in the lorazepam and 42% in the alprazolam group	Major side effects (ataxia, deliriurn, oversedation, hypotension): lorazepam n=4, alprazolam n=0; minor side effects (agitation, restlessness): lorazepam n=5, alprazolam n=6
Coccaro et al., 1990	8-week randomized, double-blind comparison trial	Efficacy of haloperidol, oxazepam, and diphenhydramine in the treatment of agitation. As outcome measures clinician ratings of agitated behavior and ADL were assessed.	Haloperidol and diphenhydramine showed a not significant greater clinical efficacy compared to oxazepam on improvements in ADL and clinical ratings of behavioral agitation.	Low number of side effects – not further defined, Withdrawal from study caused by oxazepam; extrapyramidal symptoms, oversedation, agitation (n=1, for each)

Abbreviations: AD, Alzheimer’s disease; ADL, activities of daily living; BZD, benzodiazepine; i.m., intra muscular; n.d., not determined.

Table 4 summarizes study designs, methodology, and main findings of the 5 studies, which assessed the effect of BZDs for behavioral and sleep disturbances in patients with AD.

**Table 5.** Percentage of BZD Use of Patients with AD in Selected Study Populations

	Patients in Whom BZD Use Was Evaluated **N**	Patients with AD **N (%**)	Patients Treated with BZD if AD<100% **N (%**)	AD Patients Treated with BZD **N (%**)
Frequency of BZD use				
Koyama et al., 2013	1.484	n.d.	128 (8.6)	n.d.
Montastruc et al., 2013	684	684 (100)	-	58 (8.5)
Sterke et al., 2012	284^*^	n.d.	n.d.	n.d.
Wetzels et al., 2011	117	41 (35)	15 (12.8)	n.d.
Nobili et al., 2009	349	203 (58)	85 (24.3)^*a*^	n.d.
Steve et al., 2008	66	38 (57)	9 (14)	n.d.
Balfour et al., 2003	460	460 (100)	-	88 (19.1)
Lagnaoui et al., 2003	4.214	4.214 (100)	-	842 (20)
BZD effects on cognitive functions				
Rosenberg et al., 2012	230	230 (100)	-	17(7.4)
Ellul et al., 2006	224	224 (100)	-	30 (13)
Lopez et al., 1999	179	179 (100)	-	23 (13)
Sunderland et al., 1989	20	10 (50)	-	10 (50)
Larson et al., 1987	308	17 (5.5)^*b*^	13 (4.2)^*c*^	n.d.
BZD for behavioral and sleep disturbances				
Liao et al., 2012	7	6 (86)	-	6 (86)
Meehan et al., 2002	272	n.d.	68 (25)	n.d.
McCarten et al., 1995	7	7 (100)	-	7 (100)
Ancill et al., 1991	40	n.d.	40 (100)	n.d.
Coccaro et al., 1990	52	n.d.	19	n.d.

Abbreviations: n.d., not determined, -, not applicable.

^*a*^BZD use represents the co-medication to prescribed antipsychotics.

^*b*^Indicates number of AD patients of all 35 cases with Adverse Drug Reaction (ADR) causing cognitive impairment.

^*c*^Persons developing ADR causing cognitive impairment due to documented use of BZDs.

^*^Percentage of AD patients determined in another study from the same cohort.

Table 5 shows that detailed information regarding number of especially AD-patients treated with BZDs is missing in approximately 50% of studies assessing the effect of BZD in the elderly.

Lopez et al. (1999) assessed the effect of antidepressants, antipsychotic agents, and BZD (hypnotics, anxiolytics) on cognition, global functioning, and psychiatric symptoms in 179 patients with probable AD during a mean follow-up time of 4.2 years. Although only 6% of the participants took BZD, their use was significantly associated with shorter time to death (RR=1.96). The frequency of adverse drug reactions (ADRs) in outpatients with suspected dementia was studied in a prospective 1-year follow-up study (Larson et al., 1987). Patients receiving BZD were 5.9 times more likely to have ADRs such as falls and cognitive impairment compared with patients without this medication. These findings are based on the observation of 35 ADRs occurring in 308 patients within study duration. Further, 56% of the patients received BZD monotherapy; the remaining patients used BZDs in combination with other drugs that potentially cause cognitive impairment.

The acute effect of lorazepam 1mg on memory and cognitive functions was studied in a randomized, placebo-controlled trial (Sunderland et al., 1989). Patients with probable mild-to-moderate AD were compared with healthy controls using standardized neuropsychological tests 1 hour after drug administration. While both groups showed an equal level of sedation, AD patients showed predominantly attention but no further memory impairments.

### BZD for BPSD and Sleep Disturbances in AD

We reviewed 5 studies assessing the effect of BZD on behavioral and sleep disturbances. As shown in Table 4, except for one (Liao et al., 2012), all studies employed a prospective design. Two included exclusively AD dementia patients (McCarten et al., 1995; Liao et al., 2012) (Table 1).

Meehan et al. (2002) compared the effect of intramuscular lorazepam 1mg to intramuscular olanzapine or placebo in a double-blind, randomized study of 272 patients with AD or vascular dementia (Table 4). A significant improvement on acute behavioral disturbances (ie, agitation) 2 hours after an intramuscular administration of lorazepam and olanzapine was reported on the PANSS Excited Component scale compared with placebo (-8.5 and -8.7 vs -5.3). ADRs such as drowsiness and dry mouth were more common in the lorazepam group.

Another randomized double-blind study (Ancill et al., 1991) compared the daily oral administration of alprazolam with lorazepam in dementia inpatients with agitation. Treatment response, as measured by the Clinical Global Impression, was 29% on lorazepam and 42% on alprazolam without significant group differences.

Coccaro et al. (1990) examined 52 patients with dementia in an 8-week randomized, double-blind comparison trial of haloperidol (0.5–5mg/d), oxazepam (10–60mg/d), and diphenhydramine (25–200mg/d). Outcome measures included changes in behavioral disturbances and activities of daily living. Results showed modest efficacy for all 3 drugs on behavioral disturbances and a small but not significantly better effect of haloperidol and diphenhydramine compared with oxazepam on activities of daily living.

BZD effects on sleep disturbances were studied in 2 prospective studies with AD patients (McCarten et al., 1995; Liao et al., 2012). Liao et al. (2012) conducted a retrospective analysis of diurnal and nocturnal activity levels following the administration of a single dose of 0.25mg brotizolam on 7 consecutive days in 7 patients with dementia. They reported a significant increase in the incidence of reversed rest-activity patterns. The interpretation of this study is difficult because of the low number of included AD patients (n=6) and the short assessment period. In a second placebo-controlled study, McCarten et al. (1995) evaluated the effect of a nighttime dose of 125mg triazolam on 3 consecutive days on sleep and memory function. They found no effects of triazolam on either parameter.

## Discussion

Despite the common clinical use of BZD in AD patients, there is a worrisome paucity of evidence from well-designed clinical studies. BZD are frequently used in patients with AD, even though their benefit and safety are not convincingly demonstrated. We observed a high degree of heterogeneity of published studies in this systematic review. Since the majority of studies are open-label, conclusions must be considered preliminary.

### Frequency of BZD Use in Persons with AD

Based on our review, the frequency of BZD use in patients with AD varies between 8.5% and 20%, whereby a much higher prevalence cannot be ruled out as chronic use of BZD has been reported in 50% among nursing home residents, independently of reporting comorbid dementia (Bourgeois et al., 2012). We suggest that the frequency of BZD use depends on the studied population, with a higher percentage in nursing homes and special care units. The majority of studies were heterogeneous regarding patient populations and disease stages of AD. Importantly, the effect of BZD use was often evaluated in conjunction with other psychotropic co-medication such as antipsychotics, anticholinergics, or antidepressants (Nobili et al., 2009; Wetzels et al., 2011; Sterke et al., 2012; Koyama et al., 2013; Montastruc et al., 2013). In most of these studies, the indications for BZD prescriptions were not well defined. For instance, 40% of the residents in Italian Alzheimer special care units receiving antipsychotics were also treated with BZD (Nobili et al., 2009), but neither indication nor dosage were determined.

A comparably low frequency of BZD use (8.5% and 8.6%) was found in 2 studies in community-dwelling elderly in the United States and France (Koyama et al., 2013; Montastruc et al., 2013). Both prospective studies considered BZD as PIM, which may contribute to other comorbidities and impair cognitive function. PIM prescriptions were positively associated with a higher overall drug consumption and female gender. Furthermore, Balfour and O’Rourke (2003) have reported higher rates of BZD prescription in agitated AD patients with musculoskeletal pain compared with patients without pain.

In a large cohort, Lagnaoui et al. (2003) detected a prevalence of BZD use of 20% of cross-sectionally studies institutionalized patients. The frequency of BZD use in nursing homes residents varied considerably depending on the attribution of BZD to subgroups termed anxiolytic and hypnotic drugs and precluded further evaluation. Two studies found a decrease of BZD use with increasing severity of dementia (Lagnaoui et al., 2003; Steve et al., 2008). These results may reflect a rising awareness around safety concerns regarding the use of BZD in very advanced stages of AD.

### Effects of BZD on Cognitive Functions

Five studies reported a negative effect of BZDs on cognitive functions in AD patients, but a high prevalence of psychotropic polypharmacy makes it difficult to judge the effects of BZDs on cognition and behavioral symptoms. Rosenberg et al. (2012), for example, reported that all classes of psychotropic medications including antidepressants, BZD, and antipsychotics were associated with a more rapid decline of cognition in AD patients, suggesting a potentially additive effect of individual psychotropic medications. In support of this, the exclusive use of either antipsychotics or BZD was not significantly associated with the worsening of cognitive, functional, and neuropsychiatric symptoms.

Along the same lines, Ellul et al. (2007) reported that the use of antipsychotics and BZD, but not antidepressants, doubled the risk of at least one-point increase in Global Deterioration Scale over 1 year in 224 patients with AD. Unfortunately, this report lacks details regarding the doses of BZD, specific agents, duration of use, and co-medication patterns. In another longitudinal study, the use of BZD was associated with a twofold increase in mortality risk in patients with AD (Lopez et al., 1999). Antidepressants did not negatively influence outcome. Again, there is no information in this report regarding dose and duration of BZD intake, and only 6% of patients were prescribed BZD.

A high risk of falls has also been affiliated with BZD use in dementia patients as reported by Larson et al. (1987), who found a 5.9 times higher risk of falls as well as accelerated cognitive impairment in BZD users compared with nonusers. However, Sterke et al. (2012) noted a significant dose-related risk of falls in demented nursing home residents not only associated with BZD use but even slightly more frequently with antipsychotics and antidepressants.

Sunderland et al. (1989) examined the effect of a low dose of lorazepam in 10 patients with probable mild-to-moderate AD on memory and other cognitive functions. Lorazepam worsened attention but not memory. Based on these findings, the authors concluded that mild sedation with BZD is not the cause of impairment of higher cognitive functions in AD. These results need to be cautiously interpreted because of the small sample size and missing data on the long-term effects of lorazepam. Based on the available evidence, further investigations should focus on possibly also dose-dependent effect of different BZD, their specific drug metabolism, use in different stages of AD, and duration of BZD treatment.

To sum up, there is a current need to differentiate effects of specific BZD on specific outcome measures including BPSD as for instance agitation, cognitive functions, and sleep disturbances in AD patients. While some substances may be beneficial for certain aspects of BPSD, this cannot be generalized to all behavioral disturbances in the course of AD.

Besides cognitive deficits, noncognitive symptoms such as restlessness, agitation, aggression, depression, and hallucinations are common in AD dementia. These are observed in 60% to 98% in patients with dementia (Mega et al., 1996; Lyketsos et al., 2000). Despite recommendations against the long-term use of BZD in older adults to treat insomnia and agitation (Finkle et al., 2011), the use of these medications remains prevalent (Montastruc et al., 2013).

Three short-time studies have assessed the effect of BZD on BPSD symptoms using a double-blind study design (Coccaro et al., 1990; Ancill et al., 1991; Meehan et al., 2002). Results are inconsistent and suggest a positive effect of BZD in <50% of agitated patients with AD in the short term. A possible explanation for this limited efficacy may be that agitation not only represents a behavioral symptom but also a consequence of misinterpreted or unrecognized needs in persons with dementia (Livingston et al., 2014). Consequently, treatment strategies should consider individual patients’ needs and include both, pharmacological and nonpharmacological interventions. Further, our results show a high number of side effects such as ataxia and delirium (Koyama et al., 2013) associated with BZD use. Therefore, a careful verification of the indication of BZD in patients with AD must be recommended.

Among some additional studies that did not meet our predefined inclusion is a report by Calkin et al. (1997) examining the effect of clonazepam on symptoms such as agitation, anxiety, and psychotic symptoms in demented and nondemented elderly during a treatment period of up to 21 months. Treatment with clonazepam was found to have limited positive but no negative effects on behavioral symptoms in both demented and nondemented patients. Again, the interpretation of this study is limited by small sample size and a high variability in treatment duration and dosage of clonazepam. Even though these studies could not be included in this review, they provide interesting and important considerations for further trials.

There is a lack of evidence for guiding drug treatment of sleep disturbances in AD; insomnia may be the leading indication for chronic BZD use in older adults. Bourgoeois et al. (2012) studied 1730 nursing home residents and found a negative association between BZD use and dementia severity. BZD were used in a higher dosages than recommended for older adults. In addition, long-term use of BZD is reported frequently in the elderly, while most clinical trials assess primarily short-term effects. For example, 2 studies over a treatment period of 3 to 7 nights included only 14 patients, 9 of whom had a co-medication with antipsychotics or sedating antidepressants (Table 4). While Liao et al. (2012) reported an increase of reversed rest activity following the administration of brotizolam, McCarten et al. (1995) found no effect of triazolam on sleep or memory functions. There is considerable uncertainty about the risk-benefit balance in this indication supported a by recent Cochrane database review (McCleery et al., 2014).

In summary, since 1983 there are very limited valid data on the effect of BZD in the treatment of behavioral disturbances, agitation, or sleep disorders in AD. Two studies indicate some evidence for a positive effect of lorazepam in the treatment of agitation, but there is no conclusive evidence on the improvement of sleep in response to BZD.

### Limitations

Several limitations apply to this review. First, the frequent coadministration of other psychotropic drugs in many of the reviewed studies is a likely source of imprecision. Second, studies included in this review used different criteria for diagnosing dementia. Although, all reported criteria are widely accepted and established, distinct differences between NINCDS-ADRDA, DSM-IV, International Classification of Diseases 10th edition, or DSM-III criteria may limit the comparability of studies. The database restriction and our search strategy may have missed some studies that were not published in PubMed/Medline and studies obtained negative results may not have been published at all. Lastly, there may be relevant information published in languages not included in our search.

## Conclusions and Perspectives

In summary, it can be assumed that every fifth to tenth person with AD receives a BZD at least once in the course of the illness. This might reflect a clinical need to treat behavioral symptoms occurring in AD patients; however, there is little evidence on which to base guidelines and recommendations for safe and effective use of BZDs in these patients. Even though studies addressing specifically dose-related effects of BZD in healthy and demented elderly are rare, early studies with a single administration of triazolam (Greenblatt et al., 1991), for example, support the assumption of age-related differences in pharmacodynamics and pharmacokinetics of BZD in the elderly.

With the exception of acute agitation, we found no evidence for the effective use of BZD in patients with AD but a number of studies reporting negative effects. RCTs examining these effects are the exception. Furthermore, we encountered a number of methodological limiting factors in the available literature.

Therefore, future studies on the benefit/risk profile of BZD in the treatment of behavioral disturbances need to follow the same high-quality scientific standards of clinical trials as for other psychotropic medications. Until such trials become available, clinicians should be well advised to consider alternative management options. These include, for example, citalopram, which has been demonstrated to reduce agitation and caregiver distress in agitated AD patients (Porsteinsson et al., 2014). Similarly, galantamine can be used as a first-line treatment of BPSD symptoms except for prominent irritation and agitation, where risperidone is more efficient (Freund-Levi et al., 2014).

In conclusion, the currently available evidence precludes clear recommendations for an evidenced based use of BZD in patients with AD.

## Statement of Interest

Dr. Wolfgang W. Fleischhacker is supported by research grants (Otsuka, Janssen Cilag, and Lundbeck), advisory board honoraria (Lundbeck, Roche, Otsuka, Janssen Cilag, Takeda, Amgen, Teva, and Targacept), speaker honoraria (Lundbeck), Janssen Cilag, Otsuka, Roche, and Takeda), and stocks (MedAvante). There are no other conflicts of interest.
